# Periprosthetic proximal femoral fractures in cemented and uncemented stems according to Vancouver classification: observation of a new fracture pattern

**DOI:** 10.1186/s13018-020-01619-4

**Published:** 2020-03-10

**Authors:** James Karam, Paul Campbell, Shivang Desai, Michael Hunter

**Affiliations:** Gosford District Hospital, Central Coast Local Health District, Gosford, NSW 2250 Australia

**Keywords:** Hip, Arthoplasty, Periprosthetic, Fracture, Classification, Pattern

## Abstract

**Background:**

Periprosthetic fractures are increasingly encountered in hip arthroplasty. The Vancouver classification system is widely used. Little knowledge exists regarding the association of the Vancouver classification with either cemented or uncemented stems. The aim was to analyse a series of fractures and determine associations.

**Methods:**

A series of consecutive patients over 8 years was identified including only post-operative fractures of primary hip arthroplasties. Baseline and radiographic characteristics were recorded including the type of stem fixation (cemented or uncemented) and Vancouver classification. Statistical analysis was performed to determine the association of the Vancouver classification between cemented and uncemented stems.

**Results:**

A total of 172 patients were identified (84 cemented stems, 88 uncemented stems). There were 30 Vancouver A fractures (12 cemented vs.18 uncemented, *p* > 0.05), 125 Vancouver B fractures (63 cemented vs. 62 uncemented, *p* > 0.05) and 17 Vancouver C fractures (9 cemented vs. 8 uncemented, *p* > 0.05). The Vancouver B2 fracture occurred most frequently (*N* = 95; 44 cemented vs. 51 uncemented, *p* > 0.05) and consists of four distinct fracture patterns: the previously described comminuted ‘burst’, clamshell and spiral patterns and the newly observed ‘reverse’ clamshell. The burst and spiral fracture patterns are significantly associated with cemented stems, and the clamshell pattern is significantly associated with uncemented stems.

**Conclusions:**

Vancouver A, B and C fractures occur equally in cemented and uncemented stems. Awareness of four distinct Vancouver B2 fracture patterns, including the newly observed reverse clamshell, will aid surgeons in predicting stem instability.

## Background

Periprosthetic proximal femoral fractures are an increasing problem in hip arthroplasty. The reported frequency is 3.5% at 20 years after primary implantation and is increasing in conjunction with rising rates of arthroplasty [1, 2]. Fractures occur intraoperatively, often in association with uncemented stems [3]. More commonly, fractures occur postoperatively secondary to falls in a frail elderly population. Despite international variations in the usage of cemented and uncemented stems, there is no clear evidence demonstrating an increased risk of fracture in one stem type over the other [4–6]. The Vancouver classification system of periprosthetic femoral fractures has widely been adopted by surgeons [7] (Table 1) and has been shown to be reliable [8–10].

**Table 1:** Vancouver classification

Vancouver classification	A: fractures of the trochanteric region; stem stable	B: fractures around or just distal to the stem	C: fractures well distal to the stem; stem stable
**Subtypes**	**AG:** fracture of the greater trochanter	**B1:** stem stable	
		**B2:** stem loose, good bone stock	
	**AL:** fracture of the lesser trochanter	**B3:** stem loose, poor bone stock	

To date, no definitive relationship has been established between fracture pattern according to the Vancouver classification and stem type whether cemented or uncemented. In particular, it is unknown whether Vancouver B2 fractures, denoting a loose stem, occur with greater frequency in one stem type over the other. The primary aim of this study was to determine the associations between fractures in cemented and uncemented stems and the Vancouver classification. A secondary aim was to investigate differences in baseline characteristics between patients sustaining periprosthetic fractures of both stem types. The proposed utility of this study will be in adding further knowledge to the nature of periprosthetic fracture patterns in hip arthroplasty.

## Methods

Ethics approval for the purpose of conducting a retrospective study was obtained from the local health district institutional review board. A consecutive series of patients in a single hospital with periprosthetic proximal fractures was identified through a search of a hospital coding database over an 8-year period from February 2011 to February 2019. Only patients with periprosthetic fractures of primary hip implants were included. Patients with intraoperative fractures, fractures of revision hips and interprosthetic fractures were excluded.

Data was recorded based on electronic documentation and analysis of digital X-rays and computed tomography scanning which was obtained in the majority of patients. Baseline details recorded included age, gender, body mass index and pre-morbid reduced mobility or carer dependence. Operative details recorded included time in months from primary implantation, indication for arthroplasty (osteoarthritis or fracture), type of stem (cemented or uncemented) and type of arthroplasty (total or hemiarthroplasty). Radiographic details recorded included the Vancouver classification, varus stem position and Dorr classification. Stem geometry (tapered or composite beam in cemented stems and straight or wedge in uncemented stems) was also recorded based on radiographic appearance. The determination of the Vancouver classification was made based on radiographic appearance and intraoperative findings in patients who proceeded to surgery. As an adjunct, a survey of plain film-only representative fracture patterns from this series was conducted amongst three orthopaedic surgeons affiliated with the study hospital but not directly involved with the study.

Statistical analysis was performed using *T* tests for continuous variables and Fisher’s exact test for categorical variables to compare a cohort of patients with cemented stems to a cohort of patients with uncemented stems with respect to baseline characteristics and the Vancouver classification. All tests were two-sided with a significance level of 0.05. Statistical analysis was performed using GraphPad Prism version 8.0.0 (GraphPad Software, San Diego, CA, USA).

## Results

### Patient numbers

A total of 1181 patients were identified after querying the hospital database. This number reflects the deliberately broad inclusion of classification codes from the trochanteric to midshaft anatomical regions to ensure unlisted periprosthetic fractures were not missed. Nine hundred seventy-eight patients with non-periprosthetic proximal femoral fractures were excluded. Of the remaining 203 patients, further exclusion was applied to 8 patients with intraoperative fractures, 6 patients with periprosthetic fractures of revision implants and 17 patients with periprosthetic fractures of hip fixation devices. After exclusions, a total of 172 patients were included in the study. All fractures were sustained after falls. Eighty-four fractures occurred in patients with cemented femoral stems, and 88 fractures occurred in patients with uncemented stems.

### Baseline characteristics

A comparison of baseline characteristics of patients in the cemented and uncemented groups is shown in Table 2. Significant differences between groups were identified with respect to age, time from initial implantation, neck of femur fracture indication for arthroplasty, primary hemiarthroplasty, varus stem placement and body mass index. In particular, almost half of the stems in the cemented group were implanted for fracture, the majority being hemiarthroplasties. There were no significant differences between groups with respect to gender, Dorr classification and pre-morbid reduced mobility or carer dependence. The majority of cemented stems were of taper design (74/84, 88.1%) with the remainder being of composite beam design (10/84, 11.9%). In the uncemented group, the majority of stems were of straight design (61/88, 69.3%) with the remainder being wedge design stems (27/88, 30.7%).

**Table 2:** Baseline characteristics

Characteristic	Cemented, *N* = 84	Uncemented, *N* = 88	*p* value
**Mean age (years)**	82.48 (SD 8.48, 95% CI 80.64–84.32, range 60–102)	78.23 (SD 9.57, 95%CI 76.70–80.76, range 40–98)	**0.0073**
**Gender (number/% male)**	39/46.43%	47/53.41%	0.4457
**Mean time from implant (months)**	68.55 (SD 80.77, 95% CI 49.15-87.95, range 1–432)	124.41 (SD 104.60, 95% CI 99.04–149.73, range 1–552)	**0.0006**
**Mean Dorr ratio**	0.52 (SD 0.08, 95% CI 0.50-0.53, range 0.37–0.76)	0.52 (SD 0.09, 95% CI 0.50–0.52, range 0.33–0.86)	0.9270
**Varus stem (number/%)**	28/33.33%	17/19.32%	**0.0393**
**Mean body mass index**	24.15 (SD 5.32, 95% CI 22.83–25.48, range 15.8–45.5)	27 (SD 4.55, 95% CI 25.88–28.13, range 18–40)	**0.0014**
**Hemiarthroplasty (number/%)**	17/20.24%	3/3.41%	**0.0006**
**Neck of femur fracture indication (number/%)**	37/44.05%	6/6.82%	**0.0001**
**Dependence on walking aid/carer (number/%)**	44/52.38%	41/46.59%	0.5419

### Vancouver classification

A comparison of the Vancouver classification of cemented and uncemented stems is shown in Table 3. There were no significant differences between cemented and uncemented stems in their association with each Vancouver classification subtype. Results of an adjunct survey of the plain film only appearance of representative fracture patterns performed by three orthopaedic surgeons indicated 100% agreement in classifying Vancouver A and C fractures and 73.14% agreement in classifying Vancouver B fractures.

**Table 3:** Vancouver classification in cemented and uncemented stems

Vancouver classification	Cemented, *N* = 84	Uncemented, *N* = 88	*p* value
**A**	12	18	0.3198
**B**	63	62	0.6080
**B1**	15	11	0.2963
**B2**	44	51	0.5399
**B3**	4	0	0.1143
**C**	9	8	0.8012

**Fig. 1:**
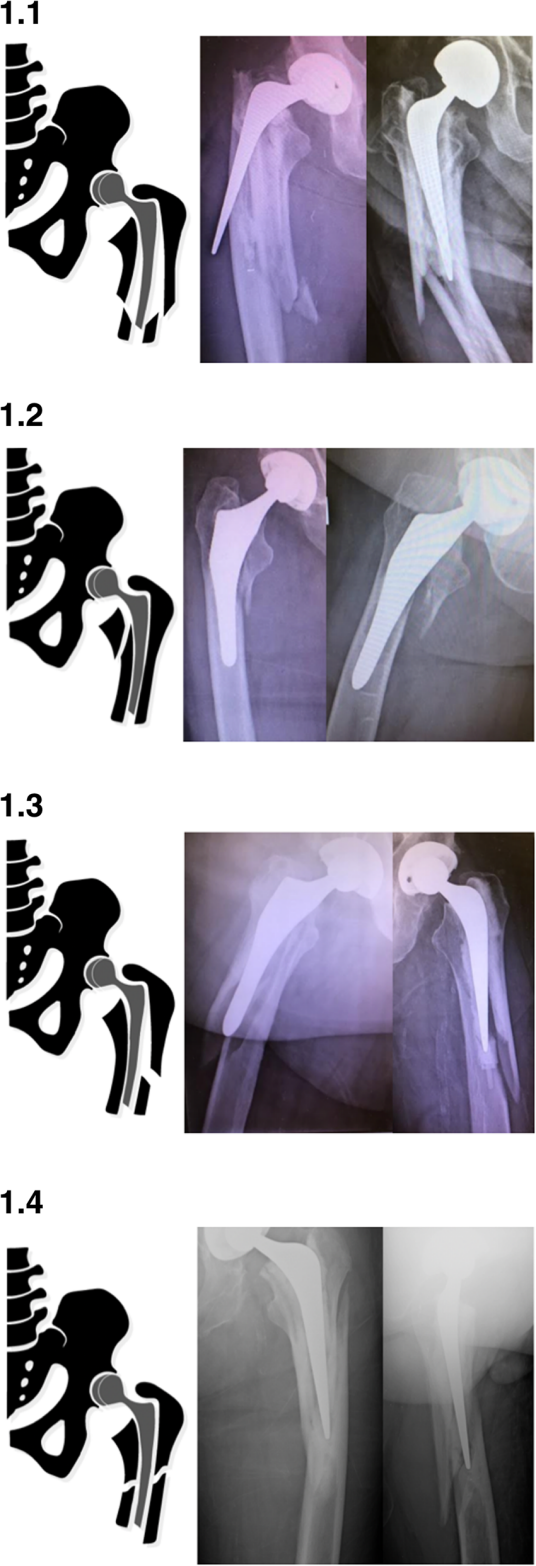
Vancouver B2 fracture patterns: **1.1** burst, **1.2** clamshell, **1.3** reverse clamshell, and **1.4** spiral

An additional observation of this study was that the Vancouver B2 classification consists of four distinct fracture patterns: the previously described comminuted ‘burst’, clamshell and spiral patterns and the newly observed ‘reverse’ clamshell pattern. Description of these fracture patterns follows in the “Discussion” section, and representative X-ray appearances from this series are shown with accompanying graphic depictions in Fig 1. The comparative association of these fracture patterns with each stem type is shown in Table 4. Burst and spiral fracture patterns were significantly associated with cemented stems whereas the clamshell fracture pattern was significantly associated with uncemented stems. The reverse clamshell pattern occurred similarly in both stems. The association of Vancouver classification subtypes including the four B2 fracture patterns listed above with respect to stem geometry (Table 5) reflected the overall trend of fracture patterns.

**Table 4:** Vancouver B2 fracture pattern in cemented and uncemented stems

Fracture pattern	Cemented, *N* = 84	Uncemented, *N* = 88	*p* value
**Burst**	18	2	**< 0.0001**
**Clamshell**	1	30	**< 0.0001**
**Reverse clamshell**	7	12	0.3336
**Spiral**	18	7	**0.0165**

**Table 5:** Vancouver classification including B2 fracture pattern according to stem geometry (number and percentage within group)

	Cemented, *N* = 84		Uncemented, *N* = 88	
Fracture pattern	Composite beam, *N* = 10	Tapered, *N* = 74	Wedge, *N* = 27	Straight, *N* = 61
**A**	1 (10%)	11 (14.9%)	4 (14.8%)	14 (23%)
**B1**	2 (20%)	13 (17.6%)	3 (11.1%)	8 (13.1%)
**B2—burst**	1 (10%)	**17 (23%)**	2 (7.4%)	0 (0%)
**B2—clamshell**	0 (0%)	1 (1.4%)	**10 (37%)**	**20 (32.8%)**
**B2—reverse clamshell**	0 (0%)	7 (9.5%)	5 (18.5%)	7 (11.5%)
**B2—spiral**	**4 (40%)**	14 (18.9%)	3 (11.1%)	4 (6.6%)
**B3**	1 (10%)	3 (4.1%)	0 (0%)	0 (0%)
**C**	1 (10%)	8 (10.8%)	0 (0%)	8 (13.1%)

## Discussion

To date, this is the largest study to directly compare the relationship of periprosthetic fractures in cemented and uncemented stems to the Vancouver classification. No significant differences were identified between cemented or uncemented stems in their association with Vancouver A, B or C fractures. The Vancouver B2 fracture occurred equally in both groups, indicating an equal rate of stable and unstable stems in periprosthetic fractures of both stems. Comparably, Fenelon et al. performed an analysis of periprosthetic fractures in cemented and uncemented stems [11]. Distinctly, a greater number of Vancouver B2 and B3 fractures were recorded in their significantly larger cemented cohort.

The Vancouver B2 fracture accounted for the greatest number of patients in either group in this study, a finding consistent with multiple other series [12–14]. An observation of this study was that the B2 fracture consists of four distinct patterns. Three have previously been described: the comminuted ‘burst’, clamshell and spiral patterns.

A highly comminuted ‘burst’ pattern in tapered cemented stems with ‘splitting’ along the cement mantle, similar to an ‘axe’, was described by Phillips et al. [15]. Supporting their observation, this fracture was significantly associated with cemented stems in this study. The highly comminuted nature of these fractures raises concerns for bone devitalisation, and these fractures often require meticulous removal of cement and bypassing with a distal bearing stem [16].

The ‘clamshell’ fracture was described by Capello et al. [17] in association with uncemented stems, a finding reflected in the results of this series. This fracture originates at the medial base of the greater trochanter and extends to the medial cortex distal to the lesser trochanter with the preservation of the lateral cortex. Widening of the calcar region and subsidence of the stem are radiographic markers of stem instability. Previous series have shown this fracture to be significantly associated with anatomical and wedge design uncemented stems [18, 19], an association supported by this study.

Grammatopolous et al. described a spiral fracture pattern in a series of periprosthetic fractures of cemented stems, often in association with a separate wedge fragment and significant comminution [20]. The significantly greater number of spiral fractures in cemented stems in this series may reflect the tendency for fractures around a tubular cement mantle to propagate in a fashion similar to native bone.

**Fig. 2:**
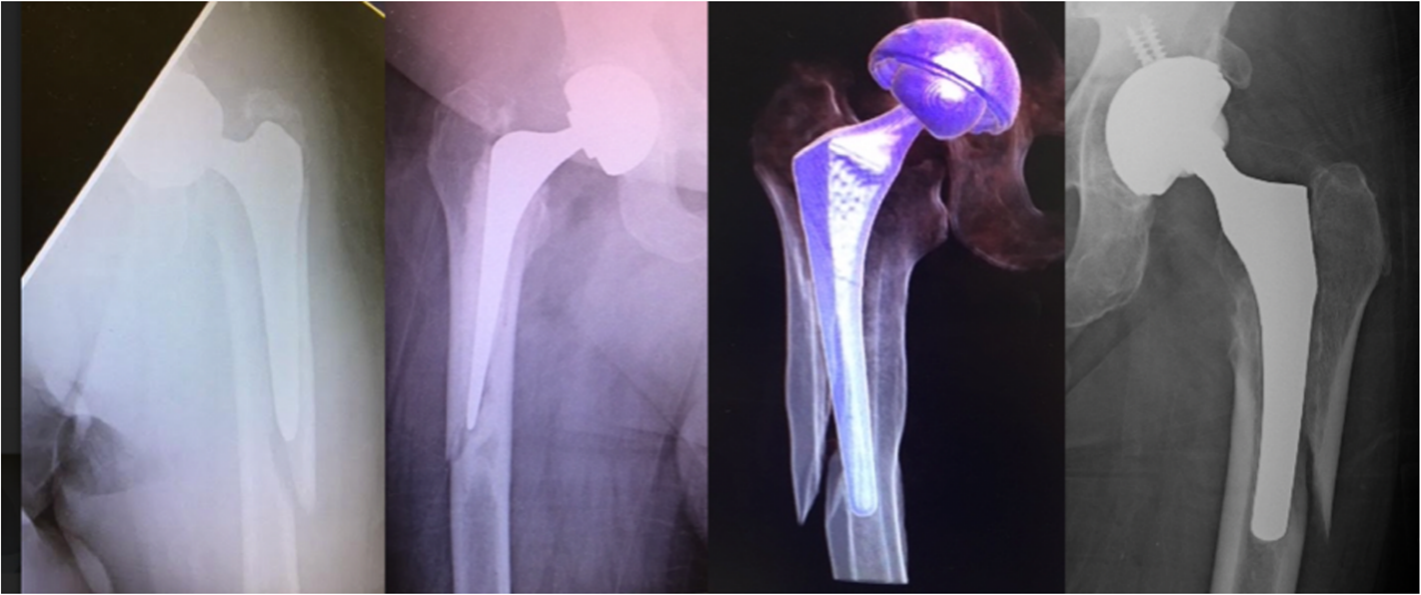
Reverse clamshell fracture pattern. In these radiographs, further examples of the reverse clamshell fracture pattern are shown in both cemented and uncemented stems displaying the typical fracture pattern involving the lateral cortex only with preservation of the medial cortex

**Fig. 3:**
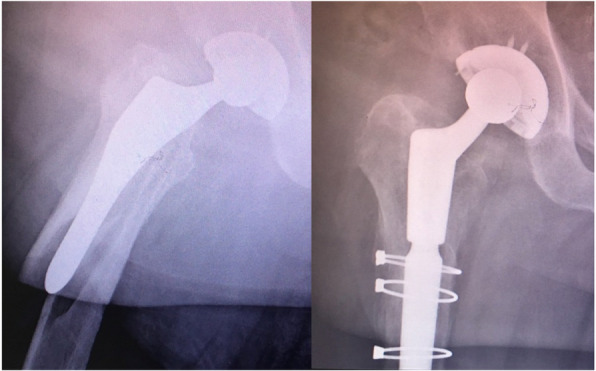
Surgical management of reverse clamshell fracture with revision arthroplasty and cerclage wire fixation

In radiographically analysing a large series of periprosthetic fractures, a consistent fracture pattern not previously described in the literature was observed. This fracture originates in the medial calcar and exits through the lateral cortex with an intact medial cortex. This fracture is named the ‘reverse’ clamshell pattern and is recognised by this study as a commonly occurring Vancouver B2 fracture pattern. This name was chosen for two reasons: the first, that it is the mirror image of the ‘clamshell’ and the second, that it behaves similarly to a reverse oblique proximal femoral fracture, with similar supero-lateral displacement of the proximal fragment from abductor pull. This fracture occurs similarly in cemented and uncemented stems, and further radiographic examples from this series are shown in Fig. 2. Although it was not the aim of this study to investigate outcomes of treatment, reverse clamshell fractures were routinely managed in this series with revision arthroplasty to a distal bearing stem with either cerclage wire or plate fixation of the proximal fracture fragment. An example of a patient treated with this approach is shown in Fig. 3 demonstrating the achievement of union.

In this series, a similar number of Vancouver B1 fractures occurred in both cemented and uncemented stems. It was observed that B1 fractures in cemented stems often occurred at the tip of the stem, and the influence of stem design and the cement mantle on fractures in this region has previously been identified [21]. It is often challenging on plain films alone to determine implant stability in undisplaced fractures of cemented stems, and computed tomography is often helpful in assessing the integrity of the cement mantle. Fracture of the cement mantle implies a loose stem and hence a B2 fracture. Keys to distinguishing between B1 and B2 fractures in uncemented stems include calcar widening, new bone-implant interface gaps and stem subsidence. Computed tomography scanning with metal artefact reduction may aide determination of implant stability, although this determination may only conclusively be made intraoperatively.

A paucity of Vancouver B3 fractures was identified in this series, and only in cemented stems. There is a variable rate of incidence of Vancouver B3 fractures in published series, and identification of strict B3 patterns may be subject to a high rate of inter-observer variability [22]. Identification of osteolysis in the setting of fracture is often a subjective assessment, particularly if prior X-rays are not available and the patient is asymptomatic prior to fracture [23]. A and C fractures occurred similarly in both groups, and these fractures likely occur independently of stem fixation and design.

The Vancouver classification system is based on the assessment of plain films alone. As intraoperative findings in patients who proceeded to surgery were factored into classification, formal validation of classification in this study was not performed. Agreement rates of 80% for this classification system have been published, with the commonest cause for inter- and intra-observer variability being distinguishing between Vancouver B subtypes [24]. This is reflected in the adjunct survey performed in this study.

It is not the intention of this study to propose a modification to the Vancouver classification system. This study supports the use of the Vancouver system in the classification of periprosthetic fractures. Regardless of fracture pattern, a B2 fracture denotes a loose stem. Each fracture pattern, including the reverse clamshell, is approached with similar principles and centred on implant stability. Although revision arthroplasty is conventionally indicated where the stem is loose, there is an increasing weight of evidence to support fixation alone in B2 fractures [25, 26].

Although a large and comparable number of patients in both cemented and uncemented groups support the observations made in this study, there were significant differences in baseline characteristics between groups. Fractures in the uncemented group occurred twice as long a period after implantation as the cemented group. This is likely explained by the uncemented cohort receiving arthroplasty at a younger age and is supported by registry data demonstrating high 10-year implant survival rates for uncemented implants [27] and a long-term series of uncemented stems demonstrating cumulative probability of fracture of 1.6% at 10 years increasing to 13.2% at 29 years after surgery [28]. The lower time from implantation of the cemented group is likely explained by the significantly greater number of patients in this group receiving arthroplasty including hemiarthroplasty for neck of femur fractures accounting for almost half of the cohort. This reflects the frailty of this population with a significantly higher age than the uncemented group. This may also explain the lower body mass index in the cemented cohort. The overall frailty of patients sustaining periprosthetic fractures is suggested by the surrogate markers of reduced mobility and carer dependence accounting for a high percentage of patients in both groups. Although a greater number of stems in the cemented cohort were placed in varus, an increased risk of fracture in varus stems has not been demonstrated in either stem type in previous studies [29, 30].

This study was limited by its retrospective design. Data collection from a single hospital limits the generalisability of results. The inclusion of patients who received arthroplasty for fracture introduced significant heterogeneity into the study population. This may have caused bias in the recording of fracture patterns in the cemented cohort of whom almost half received arthroplasty for fracture. Patients who sustain neck of femur fractures often have weaker osteoporotic bone, and the relationship of this to descriptive periprosthetic fracture patterns was not determined in this study. Overall, regardless of the indication for arthroplasty, Vancouver classification fracture patterns in either stem type were similar. Additionally, it was not the objective of this study to determine the incidence of periprosthetic fracture in cemented or uncemented stems; therefore, it was not determined for example that patients receiving cemented stems for the neck of femur fractures were at increased risk of periprosthetic fracture compared to patients receiving uncemented stems for osteoarthritis. There was a lack of standardisation of plain films performed, with a variable quality of imaging obtained reflecting the nature of imaging in an elderly injured population. Interpretation of a classification system is subject to inter- and intra-observer variability, and this was not formally assessed as intraoperative findings were factored into the determination of classification.

## Conclusions

Periprosthetic fracture types according to the Vancouver classification system occur in equal rates in cemented and uncemented stems. The rates of stable and unstable stems after fracture are therefore equal in both groups. Recognition of four distinct Vancouver B2 fracture patterns, including the newly observed reverse clamshell pattern, will aid surgeons in recognising stem instability. Future studies investigating the association of fracture patterns with treatment strategies are required to determine the clinical significance of the findings of this study.

## Data Availability

The datasets used and analysed during the current study are available from the corresponding author on reasonable request.

## Abbreviations

CI: Confidence interval
SD: Standard deviation
