# Correction: Impact of preoperative anemia on patients undergoing total joint replacement of lower extremity: a systematic review and meta-analysis

**DOI:** 10.1186/s13018-024-04765-1

**Published:** 2024-05-03

**Authors:** Fu‑Qiang Zhang, Yong‑Ze Yang, Peng‑Fei Li, Guo‑Rong Ma, An‑Ren Zhang, Hui Zhang, Hong‑Zhang Guo

**Affiliations:** 1 First Clinical Medical College of Gansu, University of Traditional Chinese Medicine, Lanzhou, China; 2 People’s Hospital of Gansu Province, Chengguan District, 204 Donggang West Road, Lanzhou, 730000 China

**Correction: Journal of Orthopaedic Surgery and Research (2024) 19:249**

10.1186/s13018-024-04706-y

The authors would like to add the missing details in the funding section. The project number (GSWSKY-2019-68) and name (Gansu Province Health Sector Project).

The updated funding is given below and the original article has been corrected.

The sentence currently reads:

This study is supported by the Natural Science Foundation of Gansu Province, China. (20JR10RA358)

The sentence should read:

This study is supported by the Natural Science Foundation of Gansu Province, China. (20JR10RA358), Gansu Province Health Sector Project, GSWSKY-2019-68.

