# Correction: A risk prediction model for postoperative recovery of closed calcaneal fracture: a retrospective study

**DOI:** 10.1186/s13018-024-04763-3

**Published:** 2024-05-08

**Authors:** Wenjing Li, Yan Wang, Zenglei Zhang, Wei Chen, Hongzhi Lv, Yingze Zhang

**Affiliations:** 1 Hebei Provincial Key Laboratory of Orthopaedic Biomechanics, Hebei Orthopaedic Research Institute, No. 139 Ziqiang Road, Shijiazhuang, 050051 China; 2 Trauma Emergency Center, The Third Hospital of Hebei Medical Fig. 7 Schematic diagram of risk scoring on the nomogram University, No. 139 Ziqiang Road, Shijiazhuang, 050051 China; 3 Rehabilitation Center, The Third Hospital of Hebei Medical University, No. 139 Ziqiang Road, Shijiazhuang, 050051 China

**Correction: Journal of Orthopaedic Surgery and Research 2023 18:612**

10.1186/s13018-023-04087-8

Following publication of the original article [1], the authors would like to correct the name and the number of one fund in the acknowledgement.

The updated acknowledgement is given below and the changes have been highlighted in **bold typeface**.

The sentence currently reads:

We thank and acknowledge the contribution of all the participants and team members. This study was supported by The National Natural Science Youth Foundation of China (Grant No. 8,210,091,792), The Hebei Natural Science Foundation (Grant No. H2015206449), and Hebei Medical Science Research Project (Grant No. 20,210,552). The corresponding author had full access to all the data in the study and had fnal responsibility for the decision to submit for publication.

The sentence should read:

We thank and acknowledge the contribution of all the participants and team members. This study was supported by The National Natural Science Youth Foundation of China (Grant No. 8,210,091,792), **Natural Science Foundation of Hebei Province (Grant No.H2021206317)**, and Hebei Medical Science Research Project (Grant No. 20,210,552). The corresponding author had full access to all the data in the study and had fnal responsibility for the decision to submit for publication.

